# Can Labelling Create Transformative Food System Change for Human and Planetary Health? A Case Study of Meat

**DOI:** 10.34172/ijhpm.2020.239

**Published:** 2020-12-08

**Authors:** Christine Parker, Rachel Carey, Fiona Haines, Hope Johnson

**Affiliations:** ^1^Melbourne Law School, The University of Melbourne, Melbourne, VIC, Australia.; ^2^School of Agriculture and Food, The University of Melbourne, Melbourne, VIC, Australia.; ^3^School of Social and Political Sciences, University of Melbourne, Melbourne, VIC, Australia.; ^4^School of Law, Queensland University of Technology, Brisbane, QLD, Australia.

**Keywords:** Animal, Agriculture, Sustainable Diet, Law, Regulation

## Abstract

**Background:** One important way to transform food systems for human and planetary health would be to reduce the production and consumption of animals for food. The over-production and over-consumption of meat and dairy products is resource-intensive, energy-dense and creates public health and food equity risks, including the creation of superbugs and antimicrobial resistance, contamination and pollution of land and waterways, and injustice to animals and humans who work in the sector. Yet the continuing and expanding use of animals is entrenched in food systems. One policy response frequently suggested by parties from all sectors (industry, government and civil society) is voluntary or mandatory labelling reforms to educate consumers about the healthiness and sustainability of food products, and thus reduce demand. This paper evaluates the pitfalls and potentials of labelling as an incremental regulatory governance stepping-stone to transformative food system change.

**Methods:** We use empirical data from a study of the regulatory politics of animal welfare and environmental claims on Australian products together with an ecological regulation conceptual approach to critically evaluate the potential of labelling as a regulatory mechanism.

**Results:** We show that labelling is generally ineffective as a pathway to transformative food system change for three reasons: it does not do enough to redistribute power away from dominant actors to those harmed by the food system; it is vulnerable to greenwashing and reductionism; and it leads to market segmentation rather than collective political action.

**Conclusion:** We suggest the need for regulatory governance that is ecological by design. Labelling can only be effective when connected to a broader suite of measures to reduce overall production and consumption of meat. We conclude with some recommendations as to how public health advocates and policy entrepreneurs might strategically use and contest labelling and certification schemes to build support for transformative food system change and to avoid the regressive consequences of labelling.

## Background

###  Animal Use in the Food System: A Challenge for Transformative Governance

The current global use of animals in the food system has significant benefits in terms of economic development and consumption of affordable meat, but is unsustainable and unjust in the longer term. Scholars, activists and policy entrepreneurs increasingly recognize that reduction and transformation of the use of animals for food production and consumption would reap a range of co-benefits.^1-6^ There have long been concerns about the ethical treatment of animals in contemporary intensive food production.^7-9^ Intensive farming of animals for food and animal feed also has negative impacts on land use, climate change, and biodiversity.^10-15^ Moreover public health analysis also calls for most high income populations to significantly reduce their consumption of animal foods, particularly processed meats, in order to reduce the risk of non-communicable diseases, including some types of cancers.^16-18^ The coronavirus disease 2019 (COVID-19) pandemic is a stark reminder of the interconnection of human, animal and environmental health.^19^ The genesis of COVID-19, and other zoonotic diseases, has been linked to habitat destruction (often for production of crops as animal feed), and expansion in wild animal farming, transport and slaughter.^20,21^ Intensive animal operations have served as epicenters for the creation of new zoonotic diseases, anti-microbial resistance and sometimes food safety breaches.^22-24^ Unfair and unsafe labor practices are also characteristic of intensive animal food production.^20,24^ Intensive animal production causes air, water and noise pollution in local areas, which impacts human and environmental health. This has unequal impacts on poor and racialized communities in both low- and high-income countries, while land clearing for animal feed crop production and grazing perpetrates injustice on indigenous and traditional communities.^25^

Food systems should be understood as food webs in which humans, animals, and ecosystems all depend on each other for ongoing survival and flourishing.^26-28^ Current industrial food production and consumption practices are exceeding the capacity of the planet to provide sustainable food for all and also create health risks for many.^11,29^ Unsustainable production and consumption of animal foods is a key contributor. Recent evidence-based reports on healthy sustainable food systems have therefore recommended (*a*) a total reduction in global animal use in the production and consumption of food; and (*b*) a transformation of intensive production and over consumption towards more ecologically sustainable methods of animal (and plant) production for food.^6,24,30-33^ Most of the burden of change will need to fall on high income countries where animal production is intensive and average consumption many times higher than the global average. In low- and middle-income countries where levels of consumption and production are below the global average, a reduction in production and consumption may not be appropriate.^6^ There are also some contexts (generally in low- and middle- income countries) where traditional and mixed cropping agroecological systems already exist that might provide appropriate models for sustainable transformation.^24,31^

Contrary to these recommendations, there is ongoing expansion in the number of animals used in food production and the intensification of their use in the Global North, and also increasingly in those countries in the Global South that host expanding intensive animal production-consumption systems.^34-36^ The economic, political and cultural power of meat production and consumption is embedded in the food system.^37-39^ The economies of some of the richest and most powerful nations and the profit of multinational corporations depend on the export of live animals, animal food products and animal feed.^40^ Many humans depend on intensive animal production for livelihoods.^41^ Cultural discourses reinforce the normality and necessity of animal protein consumption.^42,43^ The “meatification” of both individual diets and national economies is also a marker of social status and economic development.^38,44^

To achieve political, economic and cultural transformation in the global over-use of animals in the food system, scholars and scientists have suggested a range of policy tools. These include: withdrawal of subsidies for intensive animal agriculture and for the production of commodity crops for animal feed; increased state support for regenerative or agroecological farming; incentives for development and marketing of healthy alternatives to meat, especially those based on whole legumes, nuts and seeds; taxes on the consumption of meat; strict environmental and welfare standards for animal production; and advice to consumers through dietary guidelines, health advice and mandatory or voluntary food labelling.^6,41,45-51^ Most governments have not (yet) taken up these policy measures. But, one area where governance to alter food systems is more tenable is food labelling.

Food labelling is frequently proposed by experts as a first step that can begin to shift food systems in a more healthy and sustainable direction through educating consumers and motivating producers to provide more sustainable options for consumer choice.^52^ Accordingly, whenever a new challenge to healthy sustainable food systems is identified, calls for government to mandate labelling or for businesses to voluntarily adopt labelling are not far behind.^53-57^ Food labelling policy has been the channel for major policy debates about the potential risks of genetically modified organisms in the food system and the public health dangers of eating foods high in fat, sugar and salt.^58,59^ Animal welfare and related environmental and health claims on animal food products, such as “free range,” “pastured,” “humane,” “natural” and “organic” are already highly visible in many countries.^60-62^ Labelling of meat products to indicate responsible use of antimicrobials is also increasing in popularity.^63,64^ There are now calls for and some developments in the labelling of meat and dairy products to address further health and environmental concerns such as greenhouse gas emissions, water use, dependence on deforestation, and cancer risk.^47,49,65^

These calls presume labelling schemes will act as a pathway to transforming the food system over time through the following steps. The first step is that labelling will educate consumers to choose healthier and more sustainable animal food products, such as those produced via regenerative or agroecological methods or healthy alternatives to meat products.^43,66^ The second step is that labelling transparency and consumer choice will put pressure on, and incentivize, food retailers and producers to switch from a system of food production that over-exploits animals to more ecologically rational animal use and plant food production.^67^ The final step is that the impact of labelling schemes on production and consumption will instigate a broader understanding of the problems with the whole food system and create pressure for transformative change through empowered citizens, producers and whole of government action.^68^

On this view, food labelling (whether voluntary or mandatory) is a regulatory mechanism intended to influence the behavior of consumers, producers and government to transform the food system.^57,69^ To evaluate the potential effectiveness of labelling as a regulatory mechanism for reducing and transforming animal use in the food system, this paper therefore adopts a regulatory studies approach. This approach draws particular attention to the assumptions behind labelling as a pathway to transformative change (discussed further below).

###  A Regulatory Studies Approach: Ecological Regulation 

Regulatory studies scholarship is concerned with how governments and other actors use law and regulation to influence how business and markets operate.^70,71^ Law and regulation are understood as strategies in a “field of struggle” where both public and private actors seek to shape “the rules of the game” in (competitive or collaborative) interaction with other actors focused on a shared goal (here, how animal production and consumption should be regulated). Powerful actors seek to have their interests protected, whilst the less powerful seek sources of countervailing power to enable their vision to prevail.^72,73^ Regulatory studies understands regulation as a dynamic and on-going process. It is an emergent outcome of a series of contests over the field of struggle from hybrid public and private governance interactions or “regulatory networks.”^71,74-80^ The important question is not whether an issue is or is not regulated. Rather, the question is how is the issue governed, by whom, and to what effect?^77,81^ Answering this question requires empirical analytic observations. The answer also has normative implications. Regulation will be more durable and legitimate if it takes account of the concerns of all actors and recognizes the social relational aspects of regulation and compliance.^82,83^ Also, although regulation is networked, the state has a particularly important role in facilitating interventions that enable inclusion of all relevant groups.^84^

FH [Author 3] and CP [Author 1] suggest ecologically responsive regulation as an empirical and normative paradigm for how to approach regulation, considering the interdependencies between human, animal and planetary health. The ecological regulation perspective expands on regulatory networks analysis in three ways.^85,86^ First, it points out that other living species and ecosystems are actors in regulatory networks. All human and business activity is embedded in ecosystems, and hence dependent on other species and earth resources. These put real limits on how human business and society can operate in the long run, requiring effective regulation to deal with those limits. This means regulation as a policy tool should not be confined to the margins where the market fails. Rather it should work with ecosystems to ensure operation within ecological limits. From this perspective, the goal of regulating meat would not be separated from its embeddedness within global and local ecosystems but situated firmly within those systems.

Second, ecologically responsive regulation recognizes that different regulatory domains cannot be treated separately – economic, social and environmental domains are all interdependent. It is not possible to use regulation to transition to a low climate impact economy without also ensuring sufficient social provision for workers and the vulnerable to ensure a just transition.^87^ Nor is it possible to put environmental limits on human activity without addressing the short-term profit oriented incentives enshrined in competition and corporate governance law.^88^ In relation to meat, worker health and safety impacts food safety, while planetary health impacts animal and human health and vice versa, as described above.

 Third, since all regulation is always dynamic and contingent, ecological regulation recognizes that there are concurrently many ways to regulate, and that one method alone (whether labelling or a tax or a government-backed prohibition) will always be insufficient. Ecological regulation takes inspiration from environmental ecosystems where a dependence on a monoculture (where one species dominates an environment) is inherently vulnerable. Ecological regulation, then, supports diversity and plurality. Labelling alone is insufficient. Nor should regulation be aimed at achieving one perfect diet or food production practice. Plurality (of production and consumption practices; and also of policy actions) will lead to adaptability and resilience within the food and policy system.

###  Evaluating Labelling as a Pathway to Transformative Food System Change

This paper uses the ecological regulation perspective to evaluate food labelling as a pathway to transformative food system change. The question is: under what conditions does labelling as governance shift food production and consumption to more ecologically responsive outcomes, and under what conditions does it simply ‘greenwash’ destructive practices?^55,89-91^ If labelling schemes are to create food system transformation then the following assumptions would need to hold:

 First, it assumes that labelling can shift power within the food system to make food production and consumption more socially and ecologically responsive. Labelling as governance assumes that governments do not have the monopoly on regulation, and that there are opportunities for others to advocate for new socially and ecologically responsive ethics for food production and consumption. It also assumes that citizens will adopt these ethics and collectively use their consumption choices to “vote” for a different food system, in which enlightened producers and retailers create new practices of production and consumption. Thus, labelling as transformative governance redistributes power away from incumbent actors who benefit from existing practices towards other actors and alternatives.

 Second, it assumes that labelling claims accurately represent the identity, provenance and production processes for food, and that governance actors (industry, government and social movements) have the power and capacity to set and enforce standards and to hold producers and retailers accountable when labelling claims are inaccurate.

 Third, it implies that consumers can influence business and government to change production practices and regulatory systems. This in turn implies that government, social movement and expert advice will prompt citizens to move beyond consumption to political action. Politically active citizens would further educate themselves on relevant issues (through sources other than the marketing on labels), recruit others to the cause (organize) and exercise power to change the food system (through political campaigns).

## Key Messages

**Implications for policy makers**

- One of the most important ways to transform food systems for human and planetary health would be to radically reduce and substantially transform the over-use of animals in the food system. Food labelling is can contribute to this.
- Labelling has strengths as a policy tool: It can gain political buy-in from diverse actors; provide a visual cue for consumers about ethical production practices and contribute to building new norms about production and consumption which may lead to further policy action.
- Labelling has weaknesses: the standards behind the labels are frequently simplistic and reductive; labels are often misleading and subject to gaming; and labelling tends to reproduce current unsustainable consumption and production trends.
- To be transformative, labelling must be accurate, accessible to all consumers and sit within an “ecosystem” of legal and governance tools that shifts power away from unsustainable production and consumption towards sustainable, accessible and affordable food choices.
- Labelling could be improved through regulatory scrutiny that identifies misleading labelling and requires labelling claims to be consistent with the constraints of a sustainable and healthy food system.

**Implications for public**

Changing the way we use and consume animals for food is important for environmental, equity and public health reasons. Food labels can help to inform our choices about consuming animal products and send messages down the food supply chain about what we want for the future of food and for animals. This study draws on a large empirical evidence base to show when food labels help transform food systems and when they do not. Food labels are important for transforming food systems, but they have to be part of a broader regulatory approach. We identify steps that government and other regulatory actors might take to best use labelling as part of a regulatory mix for transforming the use of animals in food systems.

## Methods

This paper draws on examples from large-scale empirical research on the political economy of animal welfare and free range labelling claims on Australian egg, chicken and pig products (“the project”) in order to critique these three assumptions from an ecological regulation perspective^[1]^. The project used visual sociology to track the labelling of egg, pig and chicken products available in Australian supermarkets over time including what claims they made about animal welfare and other sustainable food system issues. It used desktop review and content analysis to track public policy and newspaper debate about animal farming issues for the last thirty years. It also used a technique [CP, Author 1] labelled ‘backwards mapping’^57,69^ to investigate the production systems and governance processes that lie behind product labelling claims, using animal welfare science review, desktop review (eg, producer, industry association and certification body websites), legal analysis, and interviews and site visits (with producers, supermarkets and key stakeholder representatives). The purpose was to understand what difference (if any) label claims make to animal welfare and sustainable food practices and governance, and how these practices and their governance have changed over time. The methodology is described in detail in previous publications.^79,92-95^

In this paper, we focus particularly on empirical findings from the project’s study of the labelling of food produced from pigs (ham, bacon and pork). Pig farming raises interconnected issues of animal welfare and environmental impacts. Sows in conventional production systems are typically kept in confined stalls and crates to breed for much of their lives, while free ranging pigs can cause significant damage to land and soils if not carefully managed, due to their rooting behavior and the large amount of waste produced.^95,96^ Pig farming is also one of the largest users of antibiotics, a source of zoonotic disease, and the World Health Organization (WHO) has classified heavily processed pig meats (ham, bacon, sausage) as a known carcinogen.^17^CP [Author 1] and RC [Author 2] have previously published on the co-optation of animal advocacy on pig welfare in corporatized sow stall free labelling,^95^ but not the broader health and sustainability challenges of the over production and consumption of meat, as we do in this paper.

 The current paper does not report substantially new empirical research. Instead it draws together and extends empirical findings from the project to advance regulatory theory in a way not done previously. The previous papers published from the project focused largely on discrete examples of industry co-optation of social movement advocacy and agroecological farming in Australia. The current paper re-examines this data to draw original insights regarding labelling as a regulatory mechanism for food systems transformation and to further elaborate ecological regulation theory.

## Results

 Using data from the projects, we show that there is some truth in each of the three assumptions above about labelling as a regulatory mechanism for food system transformation. However, the transformative potential of food labelling is limited.

###  Assumption 1: Labelling Can Redistribute Power 

The first assumption is that labelling can begin to transform food systems by redistributing power away from some actors and towards others. The dynamic, networked nature of contemporary governance does provide opportunities for strategic action on food labelling that expands the relevant voices and interests in the governance of various industry practices.^97^ In Australia, for example, there are prohibitions against misleading and deceptive forms of labelling as part of broader consumer protection law. Mandatory standards found in food law regulate the display of descriptive, nutritional, and health information on food labels. Self-regulatory instruments typically determine when terms or images regarding specific “consumer values issues” can be made on labels, such as when a label can claim a product is organic or meets certain animal welfare standards (such as “sow stall free”). Compliance with such self-regulatory instruments avoids a label being in breach of consumer law. Although, sometimes consumer law will prescribe specific meanings and standards (as occurred in Australia with “free range” eggs). Each regulatory regime that governs labelling has its own specific aims and privileges specific actors (eg, consumer protection privileges consumer choice, food law is largely concerned with health and safety risks and industry self-regulation is generally concerned with bolstering the reputation of the specific industry promulgating the scheme). Ecological regulation would require labelling to be governed in a way that addresses multiple interrelated harms and concerns.

The project demonstrated that interest in and contestation of “free range” and other high animal welfare claims on labelling did broaden public policy discourse around animal agriculture to include a broad array of government and non-government agencies representing environmental values, consumer protection, agroecology and animal advocacy concerns.^79,94^

 New labelling schemes have opened spaces for social movement activism and ecological entrepreneurship to promote more sustainable ways to produce and consume food. This includes new ecologically and socially responsive labelling and certification schemes that seek to instantiate a different relationship between humans, ecosystems and animals (eg, small scale, diverse, agroecological free range farms, co-operatives and community supported agriculture), provide for stricter animal welfare standards or re-envisage animal-free foods.

However, these more radical alternatives must compete with watered down environmental, justice and welfare standards proposed by the incumbent animal food producers and retailers.^93,95,98^ For example, the project showed that the two major supermarkets in Australia, together with Australian Pork Ltd (an industry body dominated by mainstream producers), created “sow stall free” as an industry standard displayed on labels.^95^ This standard co-opted social movement activism against factory farming of pigs into a single-issue animal welfare reform and label claim (see discussion of oversimplification in Assumption 2 below) and crowded out other possibilities such as “free range” and campaigns against industrial farming of pigs. Thus, to the consumer, the widespread availability of “sow stall free” products in the supermarket looks like an expansion of supermarket and producer responsiveness to the needs of animals. Yet, this is true only to a small degree (as shown below). The project also showed that “industrial free range” egg production emerged and became enshrined in legislation as a “greenwashed,” supermarket-friendly version of what was originally an agroecologically oriented conception of hen farming.^92^

Thus, the networked nature of labelling governance provides no guarantees that new standards will be created and, implemented that include, and are accountable to, the full range of those (humans, animals, ecosystems) harmed by agri-food systems. As the project showed, self-regulation via labelling schemes represents the further privatization of regulation, rather than a broader redistribution of power.^55^ That is, it extends property rights, via intellectual property law, into definitions of ethical production practices where none existed before. For example, in Australia “sow stall free” pork production operates via the Australian Pork Ltd’s quality assurance certification program. The certification system is the intellectual property of Australian Pork Ltd, which grants producers the right to claim on their labels that a product has been certified as “sow stall free” via a trademark for a certain fee. The two dominant supermarkets in Australia contractually require their suppliers to obtain this certification from Australian Pork Ltd (although only one supermarket emphasizes it in labelling and marketing).^95^ Thus, in practice, certification and labelling has mainly shifted power from producers to retailers and industry associations in the food supply chain.^99^ Although a web of actors (workers, consumers, animals and ecosystems) were represented in broader public discourses about the future of animal agriculture, they were largely sidelined by the new certification and labelling schemes implemented by the major supermarkets that dominate the food system.^79^ However, it also created political pressure on government animal welfare standard-setting processes, and there is a possibility that Australian Pork Ltd’s approach to what “sow stall free” means will be adopted in mandatory government regulation of pig welfare.^95^

###  Assumption 2: Labelling Claims Are Accurate and Can Be Effectively Regulated

The second assumption is that labelling claims can be appropriately regulated so that they accurately represent to the public the identity, provenance and processes used for producing food. In practice, however, the project showed that short labels designed to quickly convey meaning to a consumer are inherently simplistic and misleading; a tendency that is reinforced by the issues-based campaigning of advocacy groups, narrow responses from industry and lack of consumer knowledge and attention.^55^Label claims trumpet small or cosmetic changes (for example, pop-holes in barns for outside access that most birds do not actually use because the holes are inaccessible even when open, the barns are over-crowded and the ranges are bare and unattractive) or highlight production systems that are little different to the norm (for example, labelling intensive barn production “free to roam”).^62,92^ These small changes can “encompass,”^100^ co-opt or create a “simulacram” of alternative methods of animal production. Such tweaks in terms blur the boundaries between different production practices (for example, making intensive barn-based production appear more like smaller scale pasture-based production).^61,101^

Animal welfare labelling also overlooks the multi-dimensionality of animal well-being itself. Physical health, emotional suffering, natural behaviors and engagement with ecosystems are different dimensions of animal welfare that can be traded off against each other in commercial systems.^102-104^ Labels often emphasize just one aspect of animal welfare, typically the housing systems in which animals are kept (free range, barn or cage for eggs; sow stall free for pigs).^62,105,106^ For pigs, the focus of labelling is on just one form of confinement within an intensive indoor farming system – sow stalls (also called gestation crates) for mother or breeder pigs. This ignores other dimensions of pig welfare and the welfare of other pigs in the production system (boars and piglets/grower pigs). It also overlooks other equally important social justice and health issues raised by the production and consumption of animals in the food system. Animal justice, labor justice, health and environmental sustainability are all interconnected, as argued above.

What is true of animal welfare labelling is also true of food labelling more generally. It is rare for civil society groups with different foci – animal welfare, public health, labor justice and environmental sustainability – to form coalitions on shared interests in relation to food systems issues including labelling standards.^107-109^ Organic food labelling standards did aspire to an holistic approach but have become reductionist, industrialized and greenwashed with commercial success.^110,111^ In commercially-oriented labelling systems, the necessity to make a simple claim tends towards making the interconnectedness of food systems issues invisible in the market place and fails to build capacity to look at shared drivers of problems, and to learn to respond to inter-connectedness.^52,112^Labelling can be considered misleading to the extent that it fails to meaningfully and holistically disclose production methods or to capture the complex interconnections between health, sustainability and animal welfare, instead focusing on one favourable feature.

Consumer protection regulators in most jurisdictions can prosecute misleading claims on general law grounds, but only do so in a fraction of potential cases due to resource constraints. Moreover, such actions do not lay out constructive standards for how a private labelling scheme might deliberatively and accountably build a meaningful (and holistic) animal welfare or food sustainability standard.^61,113,114^ Rather they tend towards a least common denominator approach of whether or not a particular claim would be misleading to the ordinary consumer. This can outlaw particular claims but does not provide a positive aspirational standard for accuracy and holism in labelling claims. (We briefly discuss alternative approaches in the Discussion section below bearing in mind the competing demands of simplicity, holism and accuracy in labelling).

###  Assumption 3: Market Action Can Evolve Into Political Action 

The third assumption of labelling as a pathway to transformative food system change relies on social movements to activate individual consumers to regulate the whole food system.^55,115^ They would do this by educating themselves about food systems issues, buying relevant (labelled) products, recruiting others to the cause and turning consumer action into political action to pressure businesses and governments to transform the food system.^67^

There is some evidence to support this pathway. Bartley and co-authors conclude from their comprehensive study of the use of ethical labelling in four different areas of global trade (pulp and paper, electronics, apparel and coffee) that ‘conscientious consumerism’ can ‘be meaningful and progressive if treated as part of a repertoire of political engagement,’^89^ particularly where product labelling is used as an ‘entry point’ for hard-headed engagement with policy issues.^55,89^ There is mixed evidence from other studies about whether consumer action on environmental and social issues connects with a repertoire of political action by citizens or is merely an expression of lifestyle choice without further political resonance.^93,116^ Recent empirical research in the US has shown that adoption of animal welfare practices (such as cage free eggs) by high profile corporations (such as McDonalds) does help to create citizen support for government action to mandate cage bans for hens.^117^ The Australian project showed that ongoing contestation about how foods are labelled and marketed can keep issues alive in public policy discourse, thus keeping open the policy window for stricter regulation. It may also change citizen and political norms around what sort of regulation of animal production is appropriate. For example, the Australian free range labelling debate appears to have increased pressure to keep the policy option of banning cage production of eggs on the political agenda.^118^

Leaving it to individual consumers to change their shopping habits to make the food system sustainable and healthy is a misleading example of “lifestyle drift.” “Lifestyle drift” is a term used in the public health literature to refer to the tendency for policy to rely on individuals to take responsibility for their own health risk factors rather than addressing the social determinants of health.^119^ It suggests that consumers should be responsible for sourcing more sustainable food regardless of their financial, educational and time capacity.^115^ This ignores the many consumers who are unable to afford these products or are uneducated about or disinterested in purchasing sustainably produced foods. In practice, consumers who have little opportunity to learn much about farming systems or the systemic drivers of interconnected food justice issues are largely “educated” by simplistic labelling that actively dumbs issues down.^101,120^ Thus labelling may have the effect of dividing consumers into market segments rather than inspiring collective political action.

Labelling also divides producers into market segments – a mass market where producers undertake small, incremental improvements from time to time – and market niches (with potentially holistic, accountable, standards) that only some consumers can access. This suits retailers like supermarkets who can offer a mass market product with some (often minor, simplistic or misleading) welfare promises and some niche products with higher profit margins to maintain the custom of particularly wealthy and ethical consumers.^89,121^ It may also benefit a few high value, agroecological farmers who can obtain a price premium for highly ethical, sustainable food production. But it does not incentivize transformative change for all producers. Instead, as argued above (Assumption 2), it incentivizes misleading single-issue label claims to gain greater market share (as occurred with organic certification in the United States).^110,111^

 Thus, in the Australian project, sow stall free comprised the vast majority of the market but there was also a small market niche for truly “free range” agroecological pig products. Australia’s environmental conditions make it difficult to commercially farm pigs in a fully free range and ecologically sustainable way. The climate (particularly heat) and soil quality make most of Australia unsuitable for large-scale free range pig production. Thus, to the extent that “free range” is available, it is generally available at a high price, direct from small-scale farmers in specific locations. Yet most consumers would not be aware of this from the ready availability of “sow stall free” labelled ham and pork products available on supermarket shelves. Nor does the availability of a small market niche of expensive, sustainably produced, high welfare pork put any real pressure on large-scale production or government standards to transform the way pigs are farmed.

## Discussion

 Finally, this paper considers what steps government and civil society actors might take to maximize the possibility of labelling contributing to a transformation in the use of animals in the food system. These three points address in turn the issues highlighted above in the results, that is, the need to redistribute power, regulate accuracy, and connect market action with political action.

###  Meta-regulation of Industry Labelling and Certification Schemes

 As argued above, labelling standards for animal welfare (and other food system values) may be initiated in response to pressure to address values and interests that have hitherto been excluded by dominant production and consumption practices. Yet, these standards rarely redistribute power by institutionalizing an inclusive approach to determining those values in the governance of the scheme itself. Standards are typically reactive and non-accountable. They do not foster deliberation and diversity.

This suggests that for food labelling to be a governance mechanism with transformative capacity, such schemes should be auspiced and mandated by governments with deliberative democratic mechanisms for creating the standards behind the labels. Some government regulatory agencies (such as food standards, consumer protection, or trademark regulators) already have jurisdiction to approve food labelling standards or prosecute misleading claims. They should use these powers proactively to require deliberative democracy in private labelling initiatives. In another context CP [Author 1] has argued for this approach as a form of “meta-regulation” of self-regulated schemes.^122,123^

Decisions about labelling schemes should have mandated governance processes and expectations about who is included in standard-setting and how transparency will be ensured. When examining a proposed scheme for approval or co-developing a scheme, regulators also need to exercise the power to independently verify claims based on a range of evidence provided by various groups. Food regulators such as Food Standards Australia New Zealand and the US Food and Drug Administration do have some power to do this in narrow circumstances at present, but much more active monitoring of marketing claims is required.^113^

Deliberative processes have been used successfully in the development of animal welfare labels and organic food labels in northern European countries, such as the Netherlands and Sweden, where there is a strong tradition of joint policy-making in environmental policy, as described further below.^124,125^ Multi-stakeholder processes also present a risk of “regulatory occupation from within” by dominant actors,^92^ enabling the weakening of standards. Regardless, inclusive processes that involve multiple actors in compliance can help counter-act the influence of dominant actors within the incumbent supply chain.

 It further suggests the need to increase the accountability of the public and private agencies that implement labelling schemes, and to ensure monitoring and reviewing of labelling standards over time. Regulators could explicitly state that, when considering whether to approve or take prosecution action against labelling claims, they will take into account how those standards were created, monitored and enforced. These considerations should pay particular attention to ensuring inclusion of diverse voices and affected stakeholders.

 This meta- and inclusive approach to regulating labelling schemes should have flow-on effects on other areas of law or policy. For instance, consumer protection analyses could take account of similar considerations when examining other kinds of misleading and deceptive conduct. Government and institutional food procurement policies could also adopt similar requirements.

###  Ecological Labelling Standards

We saw above that label claims are often simplistic and misleading. Labelling approaches are needed that are based on multi-dimensional understandings of animal welfare, and that acknowledge interconnections and tensions between animal welfare and other aspects of sustainable food systems.^52,112,126^

One approach is to address the complexity of sustainable animal agriculture through the use of “tiered” labelling schemes that group a number of individual dimensions of sustainable agriculture within a general rating scheme. An example of a labelling scheme moving in this direction is the *Beter Leven* scheme in the Netherlands,^125,127^ which awards products between one and three stars based on the level of animal welfare achieved. These labelling schemes communicate a relative level of animal welfare to consumers without the use of specific, and inherently reductionist, phrases that over-simplify the issues. Rather a multi-dimensional, dynamic standard sits behind the tiers of the label which has been developed with the involvement of multiple stakeholders with civil society groups as key players. This type of scheme offers the possibility of addressing the tensions and synergies between different aspects of animal welfare.

Regardless of the exact approach used to convey information on labels, we suggest it is something to be deliberated with full openness to all concerns and interests (including animals). This suggests the need for governments, inter-governmental organizations and social movements to experiment with innovative deliberative processes for standards setting. Citizens can be invited to participate in the governance processes that help to create these standards, alongside diverse experts and stakeholder representatives (as occurs in citizen jury processes, which have an educative effect, as well as increasing accountability and legitimacy).^128,129^ These kinds of processes would educate and empower citizens and create opportunities to build shared understandings about animal welfare and sustainable food systems.

###  Contesting Misleading Labelling

 The conditions in which ethical labelling and political consumerism might serve as a “gateway drug” to political activism and deliberative democracy remain uncertain. One thing is clear: ongoing public contestation of reductionist, misleading and simplistic label claims by citizens and social movements is crucial, as has occurred in Australian policy discourse around “free range” labelling. This helps to ensure that labelling is indeed a strategy for contesting current food production and consumption practices, rather than just an aggregation of individual purchasing decisions. Labelling becomes a space in which citizens’ concerns about the interconnected injustice of animal agri-food systems are part of collective and public discourse.

 We have suggested that, in contesting label claims, attention should be paid to challenging the inclusiveness and deliberativeness of standards-making processes and emphasizing the connectedness, interdependence and vulnerability of humans to one another, animals and ecosystems. It is essential to challenge the stories and discourses that drive current animal source food systems and to create new ones that build capacity for citizens and businesses to care for animals, land, labor, health, workers and so on.

 Social movement groups would therefore be wise to prosecute misleading labelling claims and campaign for mandatory disclosures and negative warning labels on certain foods (such as health warnings on processed meats; and scan codes on animal products that provide information about welfare and environmental practices). These strategies can assist with shaming businesses (and hence changing their practices) and making contested food production practices transparent (as has occurred with mandatory labelling of trans fats, palm oil, and genetically modified foods). Hence, this approach puts issues and information in the public arena, which can then become the basis of further campaigning.

## Conclusion

 One of the most important ways to transform food systems for human and planetary health would be to radically reduce and substantially transform the way animals are used for food. Holistic policy change to transform the locked-in overuse of animals in the food system is challenging. One common response to food system problems is for expert and civil society groups to call for voluntary or mandatory labelling of food to activate consumer choice and motivate producer change for more sustainable, healthy food. This paper has used an ecological regulation approach to evaluate the potential of labelling as a first step towards transformative food system change, with a specific focus on reducing and transforming the way animals are used for food. Labelling is a popular regulatory tool, but an ecological regulatory perspective reveals the critical weaknesses that militate against labelling as a strategy that will result in systemic change. With these weaknesses identified, our analysis shows that labelling could take one of two pathways. Where labelling is narrow, captured, poorly implemented and inaccessible, it will at best create market niches of healthy, sustainable food for a privileged segment of the marketplace, while perpetuating unhealthy and unsustainable production for the vast majority. However, a second pathway is possible in which labelling enables democratic engagement with food system issues and accurately reflects the interrelated nature of these issues via holistic labelling standards. This re-orientates regulation of misleading labelling towards ensuring inclusive processes and enables actors to collectively name-and-shame misleading labels and to promote diverse business, government and citizen action to further transform the food system.

Labelling is only one part of a regulatory ecosystem that recognizes human, animal and environmental interconnectedness in food systems. Labels can only make a significant contribution to sustainable food systems if labelling schemes are anchored in broader changes in the regulatory system that allow the label to meaningfully inform and influence choices. Other elements of the ecosystem could include food policy measures mentioned above, such as the removal of subsidies for unsustainable monocultural animal agriculture and crop production for animal feed and the redirection of incentives to encourage diverse agroecological food production. In order to achieve this, it is necessary to implement a broader suite of measures, including those that ensure recognition of rights to social support and favorable working conditions for humans, and the need for humans and other living species to share healthy ecosystems for the benefit of all.^33,130^

## Acknowledgements

 The authors acknowledge previous discussions and research with Laura Boehm, Janine Curll and Gyorgy Scrinis which contributed to the research and analysis for this paper. The first author is grateful to members of the Harvard Animal Law and Policy Programme, Harvard Law School, where she was a Fellow when a first draft of this paper was written.

## Ethical issues

 Research approved by the University of Melbourne Human Research Ethics Committee. Approval Number: 1646574.

## Competing interests

 Authors declare that they have no competing interests.

## Authors’ contributions

 All authors were involved in the conceptualisation and design of various aspects of the study. CP and RC collected, analysed and interpreted the data. CP drafted the article. RC, FH and HJ reviewed and edited the manuscript for important intellectual content.

## Funding

The research reported in this paper was funded by Australian Research Council Discovery Project Grant P150102168 “Regulating food labels: The case of free range food products in Australia.” For a summary and list of outputs see https://fvas.unimelb.edu.au/research/projects/regulating-food-labels-the-case-of-free-range-food-products-in-australia/about.

## Authors’ affiliations

^1^Melbourne Law School, The University of Melbourne, Melbourne, VIC, Australia. ^2^School of Agriculture and Food, The University of Melbourne, Melbourne, VIC, Australia. ^3^School of Social and Political Sciences, University of Melbourne, Melbourne, VIC, Australia. ^4^School of Law, Queensland University of Technology, Brisbane, QLD, Australia.

## Endnotes


^[1]^ARC Discovery Project DP150102168.

